# Aggrecan deficiency does not increase susceptibility to estrogen-induced growth plate senescence

**DOI:** 10.1210/jendso/bvag163

**Published:** 2026-07-22

**Authors:** Ameya Bendre, Henrique Hadad, Lars Ottosson, Claes Ohlsson, Sara H Windahl, Ola Nilsson

**Affiliations:** Division of Pediatric Endocrinology and Center for Molecular Medicine, Department of Women's and Children's Health, Karolinska Institutet, Stockholm SE-171 76, Sweden; Department of Pediatric Endocrinology, Astrid Lindgren Children's Hospital, Karolinska University Hospital, Stockholm SE-171 76, Sweden; Division of Pediatric Endocrinology and Center for Molecular Medicine, Department of Women's and Children's Health, Karolinska Institutet, Stockholm SE-171 76, Sweden; Department of Pediatric Endocrinology, Astrid Lindgren Children's Hospital, Karolinska University Hospital, Stockholm SE-171 76, Sweden; Division of Pediatric Endocrinology and Center for Molecular Medicine, Department of Women's and Children's Health, Karolinska Institutet, Stockholm SE-171 76, Sweden; Department of Pediatric Endocrinology, Astrid Lindgren Children's Hospital, Karolinska University Hospital, Stockholm SE-171 76, Sweden; Sahlgrenska Osteoporosis Centre, Centre for Bone and Arthritis Research, Department of Internal Medicine and Clinical Nutrition, Institute of Medicine, Sahlgrenska Academy, University of Gothenburg, Gothenburg SE-413 45, Sweden; Unit of Clinical Pharmacology, Department of Pharmaceuticals, Sahlgrenska University Hospital, Gothenburg SE-413 45, Sweden; Department of Laboratory Medicine, Division of Pathology, Karolinska Institutet, Huddinge SE-141 52, Sweden; Division of Pediatric Endocrinology and Center for Molecular Medicine, Department of Women's and Children's Health, Karolinska Institutet, Stockholm SE-171 76, Sweden; Department of Pediatric Endocrinology, Astrid Lindgren Children's Hospital, Karolinska University Hospital, Stockholm SE-171 76, Sweden

**Keywords:** longitudinal bone growth, genetic growth disorders, growth plate, aggrecan, estrogen, growth plate senescence

## Abstract

In growing children, estrogen accelerates skeletal maturation, leading to advanced bone age, earlier growth cessation, and epiphyseal fusion. Children with heterozygous pathogenic variants in the aggrecan gene (*ACAN*) typically exhibit postnatal growth failure, advanced bone age, and premature growth cessation. To investigate whether accelerated skeletal maturation in aggrecan deficiency reflects increased sensitivity to estrogen-induced growth plate senescence, we used a mouse model carrying a heterozygous truncating variant in *Acan* (*Acan*^+/−^). Consistent with the human phenotype, these mice display postnatal growth failure and early growth cessation. Seven-week-old ovariectomized (OVX) wild-type and *Acan*^+/−^ mice received weekly estradiol cypionate (0.15 µg/g) or vehicle treatment for 4 weeks. As expected, OVX reduced and estradiol increased uterine weights in both genotypes. In wild-type mice, estradiol treatment significantly reduced tibial growth and several structural markers of growth plate function. In *Acan*^+/−^ mice, fewer parameters were significantly affected by estradiol treatment. Importantly, we found no evidence that aggrecan deficiency increases susceptibility to estrogen-induced growth plate senescence. These findings challenge the hypothesis that accelerated skeletal maturation in *ACAN* deficiency reflects heightened estrogen sensitivity and instead point to intrinsic abnormalities of growth plate cartilage as a driver of premature growth cessation.

In humans, longitudinal bone growth is primarily driven by endochondral ossification occurring at specialized cartilage regions termed *growth plates*. At the growth plate, growth is primarily driven by chondrocyte proliferation, extracellular matrix production, and cellular hypertrophy in a well-coordinated process. Hypertrophic chondrocytes ultimately play a crucial role in the remodeling of the cartilage into bone, resulting in net bone elongation (1). With increasing age, there is a dramatic decline in rate of growth, and this functional decline is associated with structural involution of the growth plate. In humans, the decline in growth rate is briefly interrupted by a pubertal growth spurt but then continues until growth ceases and epiphyseal fusion follows (2). This program of functional and structural decline with age has been termed *growth plate senescence* (3, 4). However, cultured resting zone chondrocytes from fetal and 16-week-old rabbits have a similar proliferative capacity indicating that classic cellular senescence cannot explain growth plate senescence (3).

Estrogen accelerates bone maturation, leading to earlier growth cessation and epiphyseal fusion (5-7) primarily mediated by estrogen receptor 1 (Esr1) (8). How Esr1 signaling regulates epiphyseal fusion is not completely understood. Previous evidence from rabbits show that estrogen does not directly cause fusion but instead accelerates the decline in functional and structural parameters (3, 7) leading to earlier fusion. Additionally, estrogen irreversibly decreases the number of resting zone chondrocytes in the growth plate, possibly providing a cellular mechanism for the hastening of growth plate senescence and epiphyseal fusion (9).

Heterozygous mutations in the aggrecan gene (*ACAN*) cause isolated short stature, typically with advanced bone age and early growth cessation, and are associated with early-onset osteoarthritis and intervertebral disc disease (SSOAOD) (9, 10). In this condition, growth cessation frequently occurs during early puberty, possibly suggesting increased sensitivity to estrogen-driven acceleration of growth plate senscence (9, 10). We recently characterized the growth phenotype of aggrecan-deficient mice using a mouse model harboring a 7-bp heterozygous deletion in exon 5 of the aggrecan gene (*Acan*^cmd^/NKruJ, hereafter referred to as *Acan^+/−^*). Similarly to humans with *ACAN* mutations, *Acan^+/−^* mice showed postnatal growth failure with early decline in structural parameters (11).

To test whether the advanced growth plate senescence of aggrecan deficiency is due to increased sensitivity to estrogen, we used this mouse model of SSOAOD (11). Ovariectomized (OVX) wild-type and *Acan*^+/−^ mice were treated with weekly injections of estradiol cypionate or vehicle for 4 weeks. We then assessed the effects of estrogen treatment on functional and structural markers of growth plate senescence.

## Materials and methods

### Animals

All experimental animal procedures were approved by the Stockholm regional animal ethical committee (#18938-2023). The cartilage matrix deficiency mouse (cmd) harbors a 7-bp microdeletion in exon 5 of the aggrecan gene (12). Homozygous mice have underdeveloped tracheal cartilage development and die at birth due to respiratory failure. Heterozygous mice are born at a normal size but develop postnatal growth failure (13, 14). The strain used in the present study was obtained from Jackson laboratory (STOCK *Acan*^cmd^/NKRUJ, #010522) and housed at Karolinska Institutet Experimental Animal Facility (AKM). In total, 48 female wild-type and heterozygous cartilage matrix deficiency (*Acan^+/−^*) mice were used in the experiments. Sample size (8 animals per treatment group and genotype) was based on previous published papers.

The animals were housed in cages (6 animals per cage) with environmental enrichment, maintained at a temperature of 24 ± 2 °C under a controlled 12:12 hours light/dark cycle, humidity (50% ± 10%), with ad libitum access to food and water.

### Ovariectomy surgeries and estradiol supplementation

For the surgeries, 6-week-old wild-type (WT) and *Acan*^+/−^ female animals (8 animals per treatment group and genotype) were randomly assigned to either sham surgery or bilateral ovariectomy surgery under inhalation anesthesia with isoflurane. Animals were treated with analgesic buprenorphine (Temgesic, 0.1 mg/kg, s.c.) once before surgery and twice daily for 2 days after surgery, to provide postoperative pain relief. Animals were allowed to recover for 1 week postsurgery. Starting at 7 weeks of age, animals were randomly assigned to receive either β-estradiol 17-cypionate (Sigma Aldrich, USA, 0.15 µg/g, s.c. [E2]) or vehicle solution Miglyol 812 (Caelo, Germany, s.c. [Veh]), once per week, for 4 consecutive weeks. Seven days after the last treatment dose, animals were euthanized and sampled for downstream analysis. Growth measurements were performed on euthanized animals using a vernier caliper. Body weight, nose-rump length, tail length, and tibia and femoral length were measured. Total body length was calculated by adding nose-rump length and tail length. Uteri were isolated and weighed. Tibiae and femurs were collected and processed for histological analyses and assessment of cell proliferation.

### Sample preparation and histology

Extracted tibiae were fixed in 4% phosphate buffered formaldehyde solution (overnight 4 °C), decalcified for 3 weeks in decalcification solution (15% EDTA, 0.5% PFA, solution replaced 3 times per week), then dehydrated and embedded in paraffin. Six-µm thick sections were used for Masson's trichrome staining and 5-ethynyl-2′-deoxyuridine (EdU) labeling.

### Growth plate histomorphometry

Quantitative histomorphometry was performed on Masson's trichrome stained proximal tibial growth plate sections. High resolution images of the samples were obtained using the Panoramic MIDI II 2.0.5 slide scanner (3D Histech, Hungary) and designated parameters were measured by using the CaseViewer software v2.2.0 (3D Histech, Hungary). All measurements were made in the central two-thirds area of the growth plate sample. Seven to eight animals per treatment group and genotype were analyzed. For each animal and parameter, averages were calculated from 15 individual measurements across 5 independent sections.

Growth plate histomorphometry was performed by a blinded researcher (A.B.) as previously described (11, 15), with minor modifications. Briefly, the number of resting zone chondrocytes were measured per 1000 µm of growth plate width. Resting zone was defined as starting at the lower margin of secondary ossification center and continuing downwards up to the flat doublet chondrocytes, indicative of the beginning of proliferative columns. Proliferative column density was calculated by counting the number of proliferative columns within 1000 µm of growth plate width. Hypertrophic chondrocytes were defined as cells with height ≥ 10 µm. Terminal hypertrophic cell was defined as the last intact hypertrophic chondrocyte in a column before metaphyseal blood vessel invasion. For height of terminal hypertrophic chondrocyte, the height of lacunae was measured as it reflects the actual height before cells shrink during tissue processing.

### EdU labeling and cell proliferation analysis

Animals were injected with EdU intraperitoneally twice (50 mg/kg, 4 and 2 hours before sacrifice). EdU staining was performed on paraffin embedded sections of proximal tibial growth plate by using Click-IT Alexa-Fluor 488 imaging kit (Invitrogen). Briefly, samples were deparaffinized and permeabilized by incubating with 4% Triton X-100 and followed by incubation in Click-IT EdU reaction buffer containing Alexa-Fluor 488 azide. Samples were then counterstained with Hoechst 3342 for nuclear staining. Samples were imaged using standard fluorescence microscopy and analyzed in ImageJ software. The number of EdU-labeled cells was quantified in the central two-thirds region of the growth plate and the EdU labeling index was calculated by dividing the number of EdU-positive cells by the total number of proliferative columns present in the region analyzed. Four to five animals per group were analyzed. For each sample, averages were obtained from measurements across 3 different sections.

### Serum sample measurements

To determine the estrogen levels produced by the estradiol cypionate (Sigma Aldrich, USA, 0.15 µg/g, s.c.) treatment, serum estradiol was quantified in blood samples obtained 7 days after the last injection in OVX mice receiving Veh or E2. Estradiol concentrations were measured using a high-sensitivity, highly sensitive and specific gas chromatography–tandem mass spectrometry (16).

### Matrix quantification

Matrix quantification was performed as described previously (11). Briefly, extracellular matrix (percentage area) was assessed using Image J software and associated Color Deconvolutor 2 plug-in tool. Measurements were made in the central two-thirds of the growth plate and in regions with identical dimensions. For each animal and growth plate, averages were calculated from measurements on 5 independent sections. After color deconvolution and thresholding, the percentage area containing cells was determined. Percentage area containing matrix was calculated by subtracting the fractional cell area from 1 and multiplying by 100.

### Statistical analyses

Statistical analysis was performed using Prism 11 software (GraphPad, USA). All data are presented as mean ± SEM. Tibial and femoral lengths were considered the primary outcomes in this study. Statistical analyses were conducted using two-way ANOVA. Post hoc pairwise comparisons were performed using Tukey's test to correct for multiple comparisons and to determine statistical significance between groups. *P* values < .05 were considered statistically significant.

## Results

In this project, breeding limitations made it difficult to achieve the target sample size (n = 8). Therefore, a sample size of n = 7 was used for some groups, including WT (OVX-Veh and OVX-E2) and *Acan*^+/−^ mice (SHAM and OVX-Veh). One uterus from the WT-OVX-Veh group was lost due to a surgical complication. Additionally, sample loss occurred during processing (dehydration, clearing, and paraffin embedding), resulting in the exclusion of one sample from WT-OVX-Veh, one from WT-OVX-E2, and one from *Acan*^+/−^ OVX-E2. EdU analysis was performed in a subset of samples, with 5 samples per group receiving EdU injection prior to euthanasia, except for WT-OVX-Veh and WT-OVX-E2, which each included 4 samples.

### Estrogen treatment decreased tibial length in wild-type, but not in *acan*^+/−^ mice

**Figure 1:**
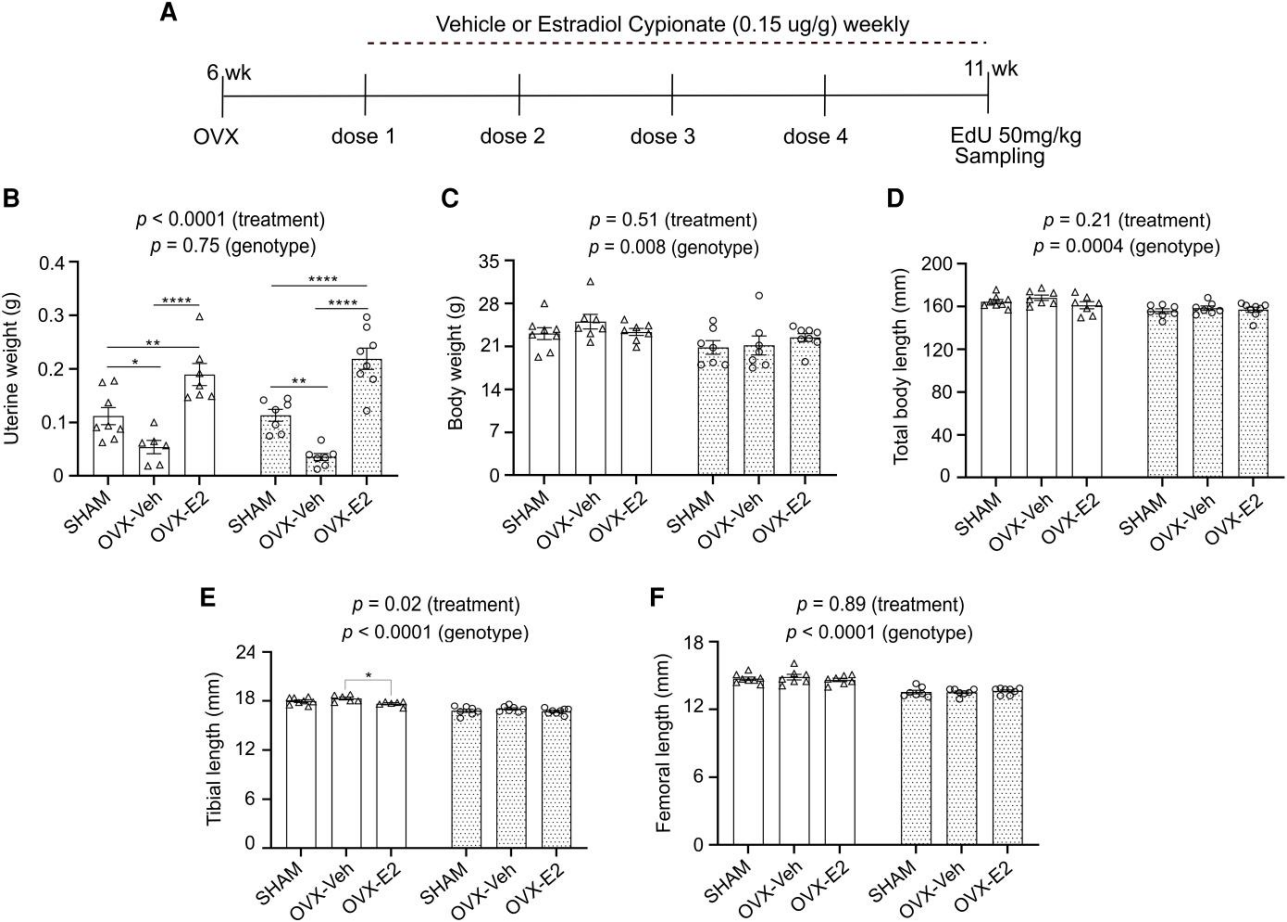
Experimental design, uterine weight, body measurements, tibial and femoral length. Effects of genotype and treatment was assessed by two-way ANOVA followed by pairwise comparisons and correction for multiple comparisons using Tukey's test. (A) Schematic representation of the experimental timeline showing the timing of surgeries, administration of estradiol cypionate (0.15 µg/g) or vehicle, and sample collection. (B) Uterine weight in wild-type and *Acan*^+/−^ mice for each experimental group. (C) Body weight in wild-type and *Acan*^+/−^ mice for each experimental group. (D) Total body length in wild-type and *Acan*^+/−^ mice for each experimental group. (E) Tibial length in wild-type and *Acan*^+/−^ mice for each experimental group. (F) Femoral length in wild-type and *Acan*^+/−^ mice for each experimental group. Open bars, Wwld-type; Patterned bars, *Acan*^+/−^. *P* values above each graph indicate the main effects of genotype and treatment obtained by two-way ANOVA. Brackets and asterisks indicate significant Tukey-adjusted pairwise comparisons.

To investigate the role of estrogen in aggrecan-related growth failure, WT and *Acan*^+/−^ female ovariectomized mice received weekly subcutaneous injections of vehicle (OVX-Veh) or estrogen cypionate (OVX-E2) for 4 weeks before sample collection (Fig. 1A). Estrogen treatment of ovariectomized mice produced physiologic serum estradiol trough levels (mean 17.6 pg/mL; range 12.0-22.2 pg/mL), comparable to those observed in intact female mice during the estrus cycle (16). These findings confirm sustained E2 exposure throughout the treatment period in estrogen-treated animals. In contrast, estradiol levels were undetectable in the vehicle-treated groups. As expected, uterine weights (Fig. 1B) were found to be significantly decreased in ovariectomized wild-type (0.1119 vs 0.0540 g, SHAM-Veh vs OVX-Veh, *P* = .04) and *Acan*^+/−^ mice (0.1133 vs 0.0354 g, SHAM-Veh vs OVX-Veh, *P* = .004) and increased with estrogen treatment compared to vehicle, in both wild-type (0.0540 vs 0.1894 g, OVX-Veh vs OVX-E2, *P* < .0001) and *Acan*^+/−^ mice (0.0354 vs 0.2190 g, OVX-Veh vs OVX-E2, *P* < .0001), indicating that ovariectomies as well as the estrogen administration were adequate. No statistically significant differences in body weight (Fig. 1C) or total body length (Fig. 1D) were detected between groups (*P* = 0.37 and *P* = .46, respectively). Also, femoral length measurements (Fig. 1F) did not show any difference among treatment groups across wild-type and *Acan*^+/−^ mice (*P* = .50); however, the tibial length (Fig. 1E) was reduced in wild-type mice OVX-E2 as compared to OVX-Veh (17.6 vs 18.3 mm, *P* = .02), but interestingly not in *Acan*^+/−^ mice (16.7 vs 17.2 mm, *P* = .36).

### Estrogen decreased growth plate height and heights of proliferative zone and hypertrophic zone in wild-type, but not in *Acan*^+/−^ mice

**Figure 2:**
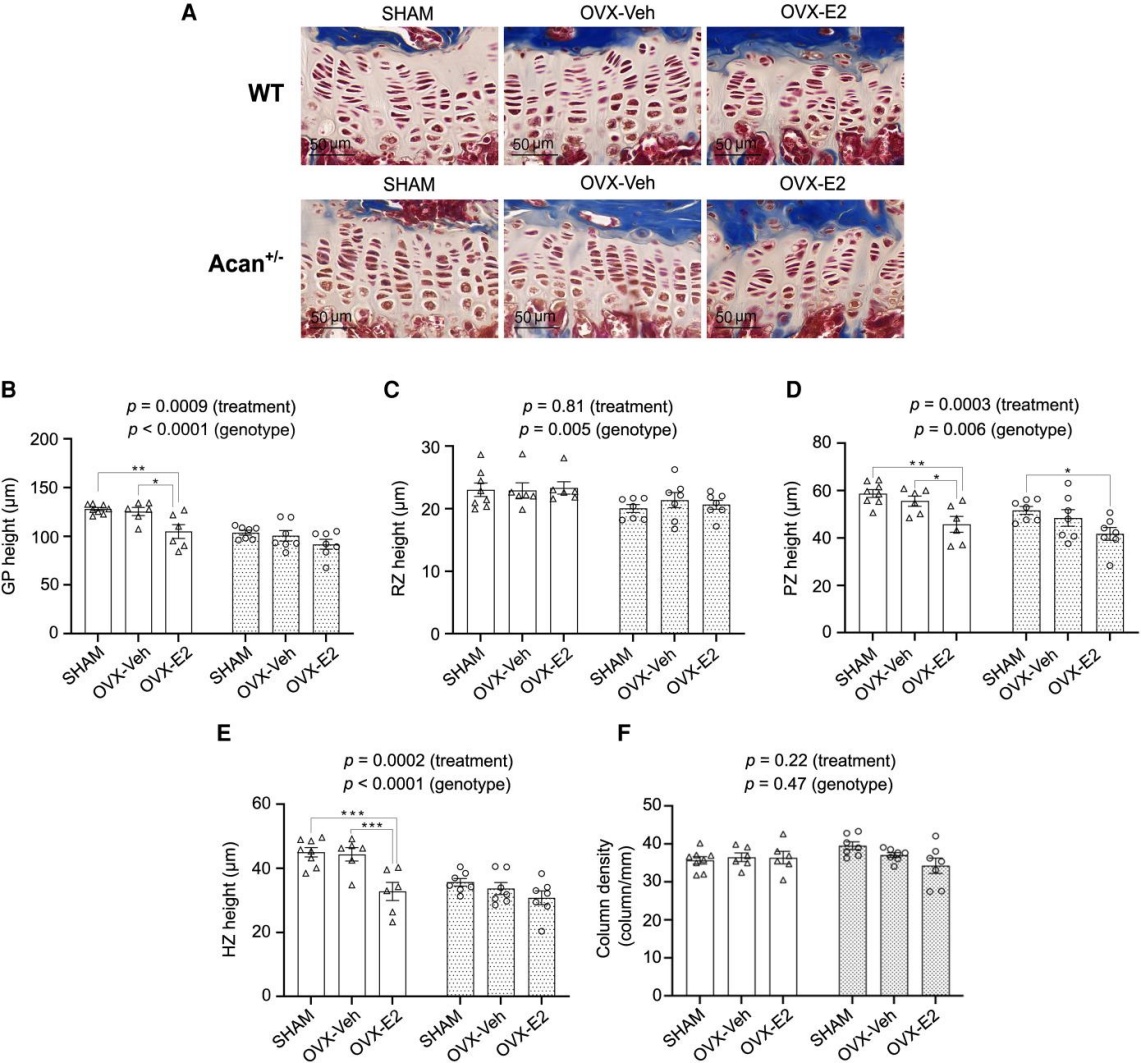
Structural parameters of the proximal tibial growth plate across experimental groups. Quantitative histology of growth plate structure was performed on Masson's trichrome stained sections of the proximal tibial growth plate. (A) Representative histological images of proximal tibial growth plates from wild-type and *Acan*^+/−^ sham operated (SHAM), vehicle-treated ovariectomized (OVX-Veh), and estrogen-treated ovariectomized (OVX-E2) female mice. (B) Growth plate height (µm). (C) Resting zone height (µm). (D) Proliferative zone height (µm). (E) Hypertrophic zone height (µm). (F) Column density (number of columns per mm). Open bars, wild-type; Patterned bars, *Acan*^+/^. *P* values above each graph indicate the main effects of genotype and treatment obtained by two-way ANOVA. Brackets and asterisks indicate significant Tukey-adjusted pairwise comparisons.

**Figure 3:**
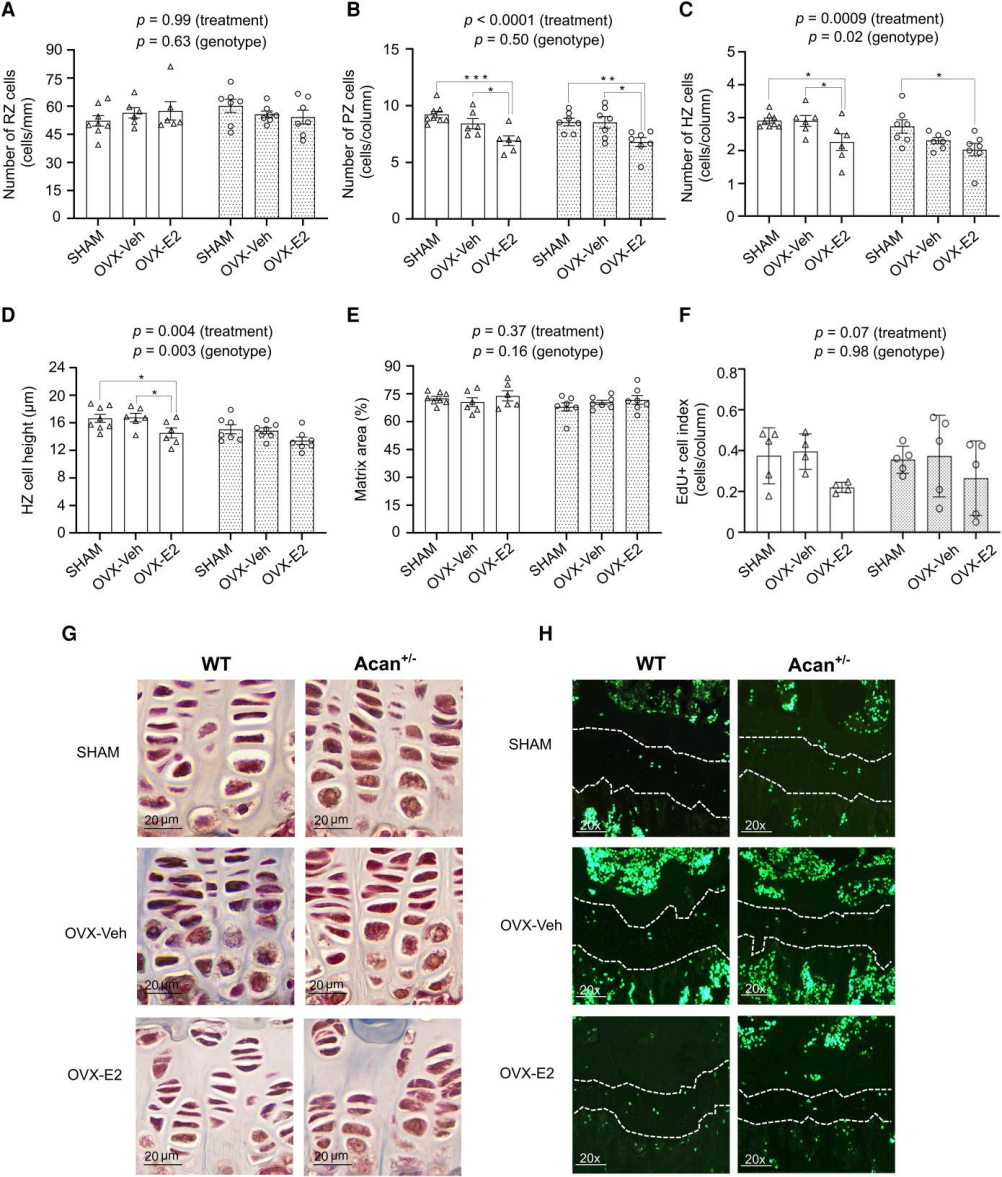
Structural and functional parameters of the growth plate. Quantitative histology of growth plate structure was performed on Masson's trichrome stained sections of the proximal tibial growth plate. (A) Number of resting zone cells per mm. (B) Number of proliferative cells per column. (C) Number of hypertrophic cells per column. (D) Terminal hypertrophic cell height (µm). (E) Percentage of matrix area. (F) EdU^+^ cell index (number of EdU^+^ cells per column). (G) Representative histological images stained with Masson's trichrome showing differences between experimental groups in wild-type and *Acan*^+/−^ mice in the proliferative and hypertrophic zones. (H) Representative images acquired after EdU staining; fluorescent green cells indicate proliferating cells. Open bars, wild-type; Patterned bars, *Acan*^+/−^. Overall ANOVA *P* values are shown above each graph. Brackets and asterisks indicate significant Tukey-adjusted pairwise comparisons.

Estrogen-treated, ovariectomized wild-type mice exhibited significant structural alterations in the growth plate, whereas aggrecan-deficient growth plates showed only mild morphological changes of lesser magnitude (Fig. 2 and 3). Growth plate height (Fig. 2A) was decreased by estrogen treatment compared to vehicle and SHAM control in wild-type mice (Fig. 2B; 104.8 vs 125.4 µm, OVX-E2 vs OVX-Veh, *P* = .01; 104.8 vs 127.8 µm, OVX-E2 vs SHAM, *P* = .002). In contrast, no significant changes in total growth plate height were detected in E2 treated *Acan*^+/−^ mice (Fig. 2B; 91.8 vs 103.7 µm, *P* = .15; 91.8 vs 100.5 µm, *P* = .35). Resting zone height differed by genotype (Fig. 2C; *P* = .008), whereas no significant main effect of treatment was detected. Estrogen treatment reduced the height of proliferative (Fig. 2D, 45.8 vs 55.5 µm, OVX-E2 vs OVX-Veh, *P* = .04; 45.8 vs 58.8 µm, OVX-E2 vs SHAM, *P* = .002, respectively) and hypertrophic zones in wild-type mice (Fig. 2E, 32.80 vs 44.36 µm, *P* = .001; 32.80 vs 45.03 µm, *P* = .0002, respectively). In contrast, estrogen-treated *Acan*^+/−^ mice only showed reduction in proliferative zone height compared to SHAM (Fig. 2D, 41.73 vs 51.63 µm, *P* = .02). However, this decrease was not statistically significant in OVX-E2 *Acan*^+/−^ growth plates compared to OVX-Veh treated group (Fig. 2D, 41.73 vs 48.46 µm, *P* = .15). Estrogen-treated *Acan*^+/−^ mice showed a slight decrease in hypertrophic zone height compared to vehicle and SHAM controls, but it was not statistically significant (Fig. 1E, 30.84 vs 33.68 µm, *P* = .54; 30.84 vs 35.66 µm, *P* = .18, respectively). No significant effect of estrogen treatment on column density was detected in either wild-type or Acan^+/−^ mice (Fig. 2F).

### Estradiol differentially modulates growth plate cellular dynamics in wild-type and *Acan*^+^/^−^ mice

In addition to the morphological changes seen in the growth plate, E2 treated wild-type mice also exhibited notable cellular and functional differences. No statistically significant differences regarding the number of cells per millimeter in the resting zone were detected (Fig. 3A, *P* = .63). However, the number of proliferative cells per column were decreased in wild-type OVX-E2 mice compared to OVX-Veh and SHAM (Fig. 3B, 6.92 vs 8.42, *P* = .04; 6.92 vs 9.21, *P* = .0008). Likewise, *Acan*^+/−^ OVX-E2 mice also displayed reduced number of proliferative chondrocytes per column when compared to OVX-Veh and SHAM (6.8 vs 8.53, *P* = .01; 6.8 vs 8.56, *P* = .009). Number of hypertrophic cells per column were reduced in in wild-type OVX-E2 mice compared to OVX-Veh and SHAM (Fig. 3C, 2.25 vs 2.9, *P* = 0.03; 2.25 vs 2.9, *P* = .02). *Acan*^+/−^ OVX-E2 mice also showed reduced number of hypertrophic cells per column compared to SHAM (2.02 vs 2.73, *P* = .01) but not to OVX-Veh (2.02 vs 2.3, *P* = .43). Terminal hypertrophic cell height was significantly decreased in OVX-E2 mice compared to OVX-Veh and SHAM (Fig. 3D, 14.53 vs 16.76, *P* = .04; 14.53 vs 16.63, *P* = .04) whereas no statistical differences were seen in the *Acan*^+/−^ OVX-E2 mice vs OVX-Veh and SHAM (*P* = .2 and *P* = .12, respectively). We did not observe any striking differences for both, matrix area percentage (Fig. 3E, *P* = .16) and number of EdU-positive chondrocytes per column (Fig. 3F, *P* = .84) in both wild-type and *Acan*^+/−^ mice with or without E2 treatment. Representative images highlighting the proliferative and hypertrophic zones in wild-type and *Acan*^+^/^−^ mice, with or without estrogen treatment, are presented in Fig. 3G, and EdU-positive cells are shown in Fig. 3H.

Although several estrogen-responsive parameters reached statistical significance in wild-type mice but not in *Acan*^+/−^ mice, two-way ANOVA did not demonstrate statistically significant genotype-by-treatment interactions for these outcomes. Thus, while the findings do not support increased estrogen responsiveness in *Acan*^+/−^ growth plates, the present study does not provide sufficient evidence to conclude that estrogen responsiveness differs between genotypes.

## Discussion

In this study we used a mouse model of SSOAOD to address the hypothesis that the advanced bone age and premature growth cessation is due to an increased susceptibility to estrogen-induced growth plate senescence. Contrary to this hypothesis, we found no evidence that *Acan*^+/−^ growth plates exhibit an exaggerated response to estradiol. In ovariectomized wild-type mice, estrogen significantly decreased several senescence-related markers, including tibial growth, growth plate height, proliferative and hypertrophic zone dimensions, and terminal hypertrophic cell size. In contrast, estrogen exposure in *Acan*^+/−^ mice led only to a decrease in proliferative chondrocytes per column, with no significant effects on other markers of growth plate senescence examined in this study. Together, these findings do not support the hypothesis that advanced bone age and premature growth cessation in SSOAOD are caused by increased sensitivity to estrogen-induced growth plate senescence.

The estrogen response in wild-type mice is consistent with prior work showing that estrogen reduces growth, growth plate height, hypertrophic cell height and accelerates declines of other growth plate senescence-related parameters (7, 9, 17, 18). Several estrogen-responsive parameters reached statistical significance in wild-type but not *Acan*^+/−^ mice. However, our data should not be interpreted as evidence for impaired estrogen signaling in *Acan*^+/−^ growth plates. Also, single-cell sequencing of *Acan*^+/−^ and wild-type chondrocytes did not detect significant differences in mRNA levels of estrogen receptor alpha, estrogen receptor beta, GPR30 or their known co-regulators (11). Thus, altered expression of these signaling components does not appear to explain the diminished estrogen responsiveness in Acan^+/−^ growth plate chondrocytes.

*Acan* ^+/−^ mice exhibit reduced extracellular matrix, impaired hypertrophic expansion, and early growth cessation (11). In this regard, aggrecan deficiency appears to phenocopy several effects of estrogen exposure on the growth plate. Reduced matrix content might alter the local signaling environment and thereby exert effects on growth plate chondrogenesis similar to those induced by estrogen. For example, extra cellular matrix components regulate the distribution and signaling of Indian hedgehog (Ihh) and other paracrine factors, thereby modulating morphogen gradients in the growth plate (19, 20).

In addition, estrogen has been shown to accelerate the depletion of resting zone chondrocytes (9), Although *Acan*^+/−^ mice do not appear to have a reduced number of resting zone chondrocytes (11), it is possible that the decreased extracellular matrix content disrupts the Indian hedgehog/parathyroid hormone–related peptide (Ihh-PTHrP) signaling loop in a manner functionally similar to resting zone depletion.

These results provide new insights into the mechanisms underlying growth failure in *ACAN* deficiency. Clinically, the affected children frequently exhibit advanced bone age and reduced pubertal growth, observations that have been interpreted as suggesting heightened sensitivity to estrogen. However, the present study found no evidence that aggrecan deficiency is associated with increased susceptibility to estrogen-induced growth plate senescence. Thus, our findings do not support the hypothesis that accelerated skeletal maturation in *ACAN* deficiency is primarily driven by enhanced estrogen responsiveness. Instead, the advanced bone age and premature growth cessation observed in these patients may reflect intrinsic abnormalities of growth plate cartilage, including impaired extracellular matrix composition and disturbed chondrocyte hypertrophic differentiation. Defects in extracellular matrix composition and chondrocyte differentiation may compromise growth plate function before the onset of puberty, thereby limiting the capacity for sustained growth during the pubertal period when growth demands increase. Thus, normal pubertal estrogen exposure may reveal or accelerate the consequences of an already compromised growth plate without requiring heightened estrogen responsiveness. Nevertheless, our findings do not exclude a role for estrogen in skeletal maturation in *ACAN* deficiency, nor do they preclude beneficial effects of interventions that modify estrogen exposure, such as aromatase inhibition. Further studies will be required to determine how intrinsic growth plate abnormalities interact with pubertal hormonal changes to influence growth and skeletal maturation in *ACAN*-related short stature.

Strengths of the study include the use of a genetically relevant mouse model and clear validation of estrogen effects in wild-type controls. Limitations include the relatively small sample size, exclusive use of female mice, a single estrogen concentration, and a relatively short treatment duration. Consequently, the study was not powered to detect subtle genotype-dependent differences in estrogen responsiveness. While the present sample size was sufficient to detect relatively large genotype-by-treatment interactions, smaller differences cannot be excluded. Nevertheless, the small observed effect sizes suggest that any true differences in estrogen responsiveness between wild-type and *Acan*^+/−^ growth plates are likely modest. Additionally, differences in pubertal timing and growth plate biology between mice and humans may limit direct clinical extrapolation.

## Conclusion

Aggrecan deficiency does not appear to predispose the growth plate to enhanced estrogen-induced senescence. Instead, our findings suggest that intrinsic abnormalities of growth plate cartilage, rather than heightened estrogen responsiveness, likely contribute to the advanced bone age and premature growth cessation observed in *ACAN*-related growth disorders. These results provide new insight into the mechanisms underlying growth failure in *ACAN* deficiency and highlight the importance of extracellular matrix integrity in regulating skeletal maturation.

## Data Availability

Data will be made available on request.

## Abbreviations

E2: β-estradiol 17-cypionate
EdU: 5-ethynyl-2’-deoxyuridine
OVX: ovariectomy
SSOAOD: short stature, advanced bone age, early-onset osteoarthritis, and osteochondritis dissecans
VEH: Miglyol 812
